# Potential of hospital wastewater treatment using locally isolated *Chlorella* sp. LH2 from cocoon wastewater

**DOI:** 10.1186/s40643-024-00748-6

**Published:** 2024-04-06

**Authors:** Tu Thi Anh Le, Truong Nguyen

**Affiliations:** Faculty of Biology, Dalat University, 01 Phu Dong Thien Vuong Street, Dalat, Lamdong Vietnam

**Keywords:** Chlorine, Native agal strain, Nutrients removal, Optimization, Pathogen removal

## Abstract

*Chlorella* sp. is able to grow and transform inorganic and organic contaminants in wastewater to create biomass. In the present study, *Chlorella* sp. LH2 isolated from cocoon wastewater was able to thrive in hospital wastewater, then remove nutrients and eliminate *E. coli* ATCC 8739. The results indicated that optimal cultivation conditions of *Chlorella* sp. LH2 in hospital wastewater were pH of 8, light:dark cycle of 16:8 at 30^o^C. The inhibitory effect of chlorination on algae growth was accompanied with the chlorine concentration. BOD_5_:COD ratio of 0.77 indicated biodegradability of hospital wastewater. The untreated and treated wastewatee samples were collected to investigated the nutrient removal efficiency after 10 days. Untreated and treated results were192 ± 8.62 mg/l 23.91 ± 2.19 mg/l for BOD_5_; 245 ± 9.15 mg/l and 47.31 ± 5.71 mg/l for COD. The treated value met the required standards for hospital wastewater treatment. The removal efficiency total nitrogen and total phosphorus were 68.64% and 64.44% after 10 days, respectively. Elimination of *E. coli* ATCC 8739 after 7 days by *Chlorella* sp. LH2 was 88.92%. The results of this study suggest the nutrients and pathogens removal potential of *Chlorella* sp. LH2 in hospital wastewater for further practical applications.

**Supplementary Information:**

The online version contains supplementary material available at 10.1186/s40643-024-00748-6.

## Introduction

Clean water is a vital resource for life. Mankind has consumed a large amount of clean water and then released various contaminants into the water bodies (Bhatt et al. 2023). Water pollution is one of major threats to the human and environment (Mannacharaju et al. 2020; Maryjoseph and Ketheesan 2020; Lin et al. 2022). Water pollutants include different contamination from domestic sewage and industrial waste. Hospitals play a pivotal role in the human well being life that has required a large volume of water. However, health services with various health scenarios generate a large amount of wastewater including hospital wastewater (HWW) and biomedical waste (BMW) (Carballa et al. 2004; Bhar et al. 2022). Besides the pollutants similar to municipal wastewater, HWW is loaded variety pollutants that act as toxic substances and infectious factors (Kümmerer et al. 2000; Snyder et al. 2003; Pauwels and Verstraete 2006). HWW is characterized by high concentration of biochemical oxygen demand (BOD), chemical oxygen demand (COD), ammonia, nitrogen, phosphorus and pathogens (Hamjinda et al. 2018). The effluents also host a significant concentration of emerging contaminants (ECs) such as pharmaceutical pollutants, personal care products, endocrine disruptors that are much greater than those in domestic wastewater (Kümmerer et al. 2000; Verlicchi et al. 2015; Kumar et al. 2022). A high density of antibiotic residues in HWW is also the main limitation for biological wastewater treatment systems (Papajová et al. 2022). Nowadays, chlorination is one of the chemical disinfected strategies due to its broad spectrum of antibacterial activity, high effectiveness and low cost (Rolbiecki et al. 2022). However the disinfection by produce and free chlorine may affect to the beneficial microorganisms of wastewater treatment process (Watson et al. 2012; Ramírez-Coronel et al. 2023).

Microalgae are extensive used in wastewater treatment due to their photoheterotrophic of available nutrients in wastewater (Aslan and Kapdan 2006; Shi et al. 2007; Mata et al. 2010; Zhang et al. 2015; Chen et al. 2020). Microalgae are able to grow in many kinds of wastewater including municipal, industrial, agro-industrial, livestock and cocoon wastewater (Lau et al. 1996; Aslan and Kapdan 2006; Ansa et al. 2015; Escapa et al. 2015; Akin 2016). Microalgae have been used in wastewater treatment systems for many benefits such as reducing COD, BOD, removing nitrogen, phosphorus and heavy metals (Escapa et al. 2015; Pandey and Gupta 2022; Silva et al. 2022; Bhatt et al. 2023). Furthermore, algae-based wastewater treatment allows for reduction of toxic compounds and ECs (Maryjoseph and Ketheesan 2020; Couto et al. 2022; Saravanan et al. 2022). The microalgae based wastewater strategy has been claimed as an inexpensive, simple, and energy efficient process (Basu et al. 2014; Srimongkol et al. 2022). The biomass is generated during treatments can be used to produce biofuel as well (Mathimani and Pugazhendhi 2019).

Microalgae can be found in HWW that contains a large number of ECs, other toxic and non-toxic pollutants and pathogens (Cuellar-Bermudez et al. 2017; Maryjoseph and Ketheesan 2020; Couto et al. 2022; Samal et al. 2022a, b). Additionally, microalgae are able to switch their metabolism between autotrophs and heterotrophs, which are termed “mixotrophic” depending on the nutrient availability (García-Muñoz et al. 2017). These unique features make them a promising choice for efficient HWW treatment (Xiong et al. 2018). Previous studies have reported the capacity of many species such as *Chlorella, Scenedesmus, Chlamydomonas* to remove different parameters of wastewater (organic matter, COD, nitrogen and phosphorus) (Heredia-Arroyo et al. 2011; Hansen et al. 2020; Mannacharaju et al. 2020; Maryjoseph and Ketheesan 2020; Xu et al. 2021). Moreover, microalgae also are able to inhibit growth of pathogens by alkalizing the environment or/and competing for nutrients (Bhatt et al. 2023).

*Chlorella*, a cosmopolitan genus, is unicellular and nonmotile. *Chlorella* sp. was widely grown in different types of wastewater and remove pollutants in wastewater (Escapa et al. 2015; Mujtaba et al. 2018; Wang et al. 2018, 2021, 2022; Wirth et al. 2020; Silva et al. 2022). Environmental conditions including pH, light:dark cycle, temperature, cultivation media and toxic compounds have an influence on growth rate and removal efficiency of algae (Panahi et al. 2019; Ziganshina et al. 2022). Thus, studying the optimal growth conditions for feasible applications in wastewater systems is important.

Among many kinds of wastewater, cocoon wastewater was similar with the components of tris-acetate-phosphorus medium (a classic algal culture medium) due to being rich in nutrients including total nitrogen, carbon, macro-elements (P, K, Na, Mg, and Ca), essential trace elements (Mn, Fe, Cu, B, and Mo), and non-essential elements (Pb and As) (Deng et al. 2020; Yang et al. 2022). These elements is an ideal condition for algae growth. It is stated that various algal species isolated from cocoon wastewater are powerful in removing pollutants including toxic and non-toxic compounds (Kümmerer et al. 2000; Li et al. 2019). Even biodegradation treatments are eco-friendly and cost effective, HWW is mainly treated by chemical oxidation methods due to ECs and pathogens (Parida et al. 2022). It is therefore worth investigating more algal species that can grow and treat HWW. In this regard, this research focus on: (i) possibility of culturing *Chlorella* sp. LH2 isolated from the cocoon wastewater in HWW. (ii) the effect of pH, temperature, light:dark cycle, chlorine on microalgae growth in HWW, (iii) possibility of *Chlorella* sp. LH2 in elimination *E.coli* ATCC 8739, a model bacterial pathogen, and (iv) efficiency of nutrients removal.

## Materials and methods

### Materials

Chemicals and reagents were obtained from Fisher Scientific (Pittsburgh, PA) and Sigma-Aldrich Co. (St. Louis, Mo). *Chlorella* sp. LH2 isolated from cocoon wastewater is available from the resource unit of Dalat University, LamDong, Vietnam. Granular calcium hypocrite (Fisher Scientific) was used as source of chlorine.

### Microalgae cultivation

**Fig. 1:**
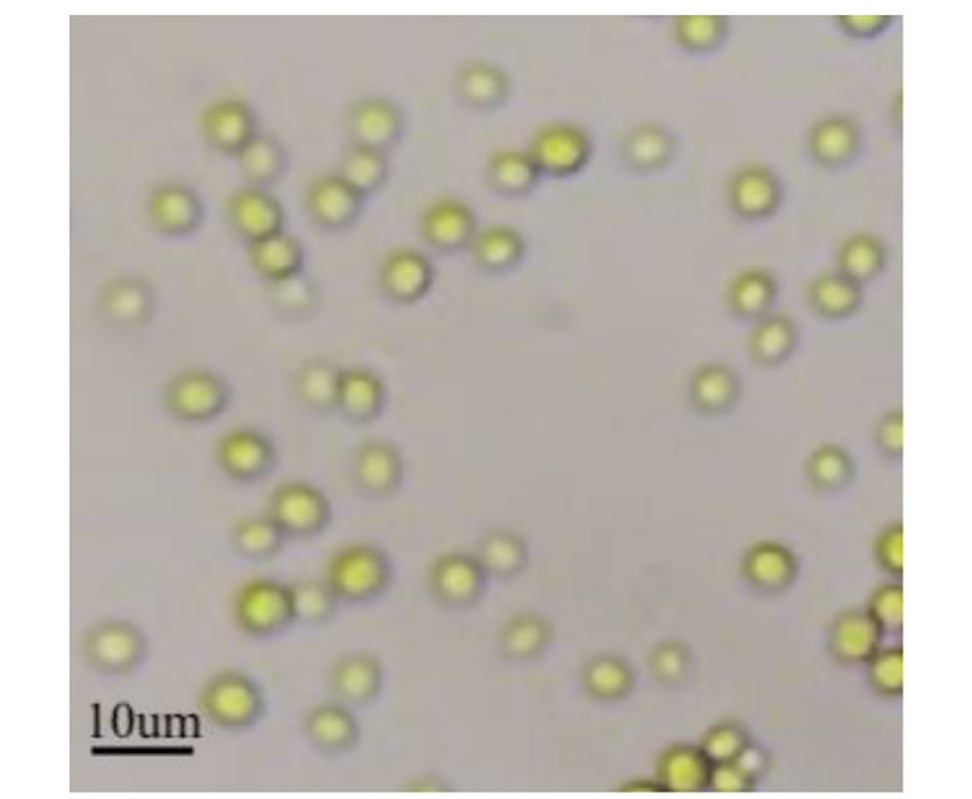
Image illustrating the morphology of *Chlorella* sp. LH2

The pure *Chlorella* sp. LH2 was isolated from cocoon wastewater has been used in this study. The size of *Chlorella* sp. LH2 was 6 ± 1 μm, having a round shape (Fig. 1). The cells were cultivated in BG11 medium. BG11 medium consists of NaNO_3_ 1500 mg/l, K_2_HPO_4_ 40 mg/l, MgSO_4_.7H_2_O 75 mg/l, CaCl_2_.2H_2_O 36 mg/l, Citric acid 6 mg/l, Trace metal solution 1 ml/l (Trace metal solution consists of FeC_6_H_5_O_7_.NH_4_OH 6 g/l, Na_2_-EDTA 1 g/l, MnCl_2_.4H_2_O 1.81 g/l, ZnSO_4_.7H_2_O 0.222 g/l, Na_2_MoO_4_.2H_2_O 0.39 g/l, CuSO_4_.5H_2_O 0.08 mg/l, H_3_BO_3_ 2.86 g/l). The culture was incubated in a closed chamber at 30 ± 2 ^o^C and shaken at 90 rpm with light intensitive of 3200 lx. The microalgae were collected by centrifugation and washed twice with distilled water for further experiments.

### Experimental setup

The ability to grow in hospital wastewater of *Chlorella* sp. LH2 was conducted in hospital, cocoon wastewater and BG11 under temperature of 30 ± 2 ^o^C and 16 h light time period. The productive of microalgae with a similar initial inoculums was determined after 7 days. The effect of temperature, pH, light:dark cycle, and chlorine were examined to identify the optimal growth conditions in sterilized hospital wastewater. *Chlorella* sp. LH2 was used at initial concentration of 0.2 g/l for all further experiments. To derive the effect of temperature on microalgae, the range of temperature was maintained at 20, 25, and 30^o^C. The favorite pH value for microalgae growth was determine at different pH levels including 6, 7, and 8. The effect of irradiation time, related to light:dark cycles of 12:12, 16:8, and 24:0 was evaluated. The effect of chlorination on the *Chlorella* sp. LH2 growth was performed at different concentrations of 0.2, 2, and 4 mg/l. The microalgae growth was measured in term of biomass concentration every 1 day. At the beginning and the end of experiments, total nitrogen, total phosphorus, BOD_5_, and COD were measure to determined removal nutrients efficiency.

In another set of experiments, *Chlorella* sp. LH2 was incubated with *E.coli* ATCC 8739 in sterilized wastewater to determine the effect of microalgae on the pathogen. *E. coli* ATCC 8739 was incubated in LB broth at 37^o^C overnight. The cells were centrifuged and washed twice with distilled water. The experiments were conducted at inocula of 4.5 × 10^5^ CFU/ml and 0.2 g/l for *E. coli* and *Chlorella* sp. LH2, respectively. The microorganisms growth was measured every 1 day in 7 days. The control consisted of *E. coli* ATCC 8739 without *Chlorella* sp. LH2 in HWW.

### Analytical methods

The algae biomass was determined by measuring OD with a spectrophotometer at 680 nm. pH was detected by a pH meter. Total nitrogen (T-N) and total phosphorus (T-P) were investigated by using a water analyzed (Pawlowski 1994). BOD_5_ and COD were measured according the Standard methods (Walter 1961). The cell density of *E.coli* ATCC 8739 was determined by dilution plating and spread plate technique (Bhatt et al. 2023).

### Statistical analysis

One-way analysis of variance (ANOVA) and t-test were performed using Excel 2011 statistical tools. A *P*-value < 0.05 was used as a criterion for significance level. ANOVA was used to determine whether growth of *Chlorella* sp. LH2 from different environmental conditions (culture media, temperature, pH, light/dark cycle and chlorine), pathogen and nutrients removal are statistical different (Fegade et al. 2013).

## Results

### *Growth of Chlorella* sp. LH2 *in different culture media*

**Fig. 2:**
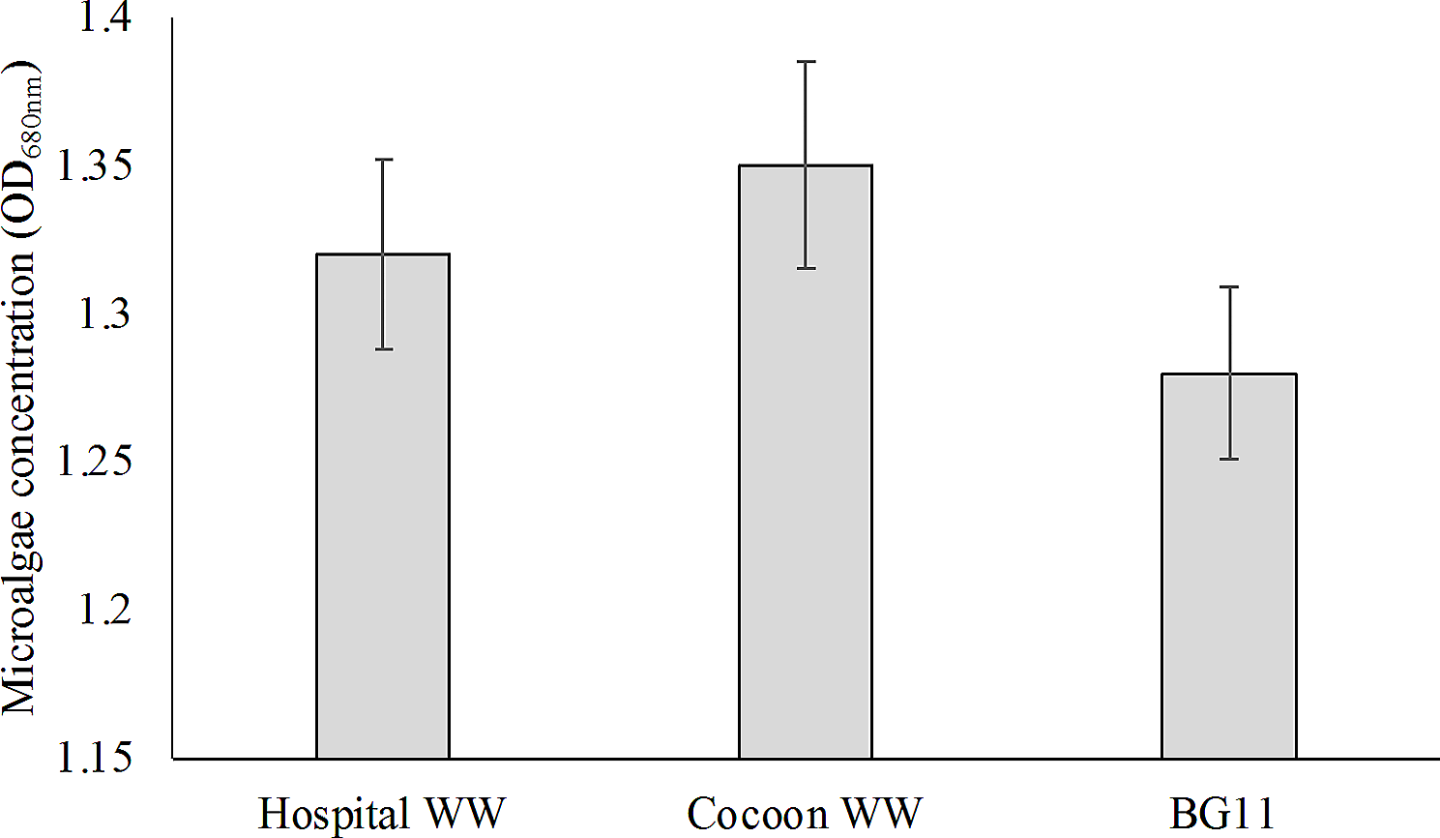
The growth of *Chlorella* sp. LH2 in different culture media (WW: wastewater)

*Chlorella* sp. LH2 isolated from cocoon wastewater was incubated in three culture media (Fig. 2). Growth was estimated through optical density (OD_680nm_). Among three culture media, the best growth was obtained in cocoon wastewater but no statistical difference compared to other culture. The yield of biomass after 7 days in HWW was no statistical difference compared to biomass obtained from cocoon wastewater and BG11 medium. The results indicated that *Chlorella* sp. LH2 survived and grew in raw HWW that is a complex of pollutants including ECs, antibiotics, and other toxic compounds.

### Effects of the temperature on microalgae production

**Fig. 3:**
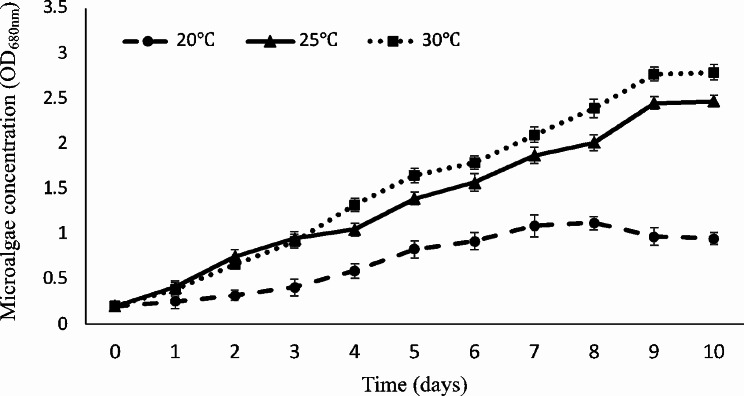
Growth curves of *Chlorella* sp. LH2 at different temperature

Fig. 3 shows the growth curves of *Chlorella* sp. LH2 in raw hospital wastewater at 20, 25 and 30^o^C. Temperature had strong influence on the cell growth. The better growth rate was observed at 25^o^C and 30^o^C compared to that at 20^o^C. After the first 3 days, the growth rate of *Chlorella* sp. LH2 at 25^o^C and 30^o^C were no statistical difference. Since the 4th day, the growth rate at 30^o^C was greater than that at 25^o^C. Physicochemical carbon dioxide availability to the microalgal cell and the metabolic processes in the cell are affected by temperature (Panahi et al. 2019; Yahya et al. 2020). Even the results are consistent with previous studies, some researches are reported the optimal growth of *Chlorella* at 25^o^C (Shi et al. 2007; Bhola et al. 2011; Fu et al. 2012; Ho et al. 2013; Bamba et al. 2015; Wang et al. 2018). This mode is likely to difference in algae strains and medium. The cell division and the accumulation of cell materials have been influenced by the different temperature (Brown 1951). The stationary phase of *Chlorella* sp. LH2 achieved at the 9th day and growth rate still remained after that. The stationary phase of microalgae at 20^o^C was observed at the 7th day but the biomass yield was lower than others (*P* < 0.05).

The less growth was observed at lower temperature (20^o^C). As the increase of temperature, the microenvironment for the enzymes involved in photosynthesis is enhanced and then cell division is promoted (Mayo and Noike 1996; Singh and Singh 2015; Bolognesi et al. 2021). Additionally, low temperature enhances the solubility of carbon dioxide (< 20^o^C) that inhibites the microalgal growth by decreasing pH level (Morales et al. 2018; Li et al. 2023). *Chlorella* sp. LH2 incubated in HWW after 10 days achieved the best growth at 30^o^C which is in the optimal range for *Chlorella* growth from 20^o^C to 30^o^C (Singh and Singh 2015; Josephine et al. 2022). Therefore, the optimal temperature of 30^o^C was used for later experiments.

### *Effects of the pH on microalgae production*

**Fig. 4:**
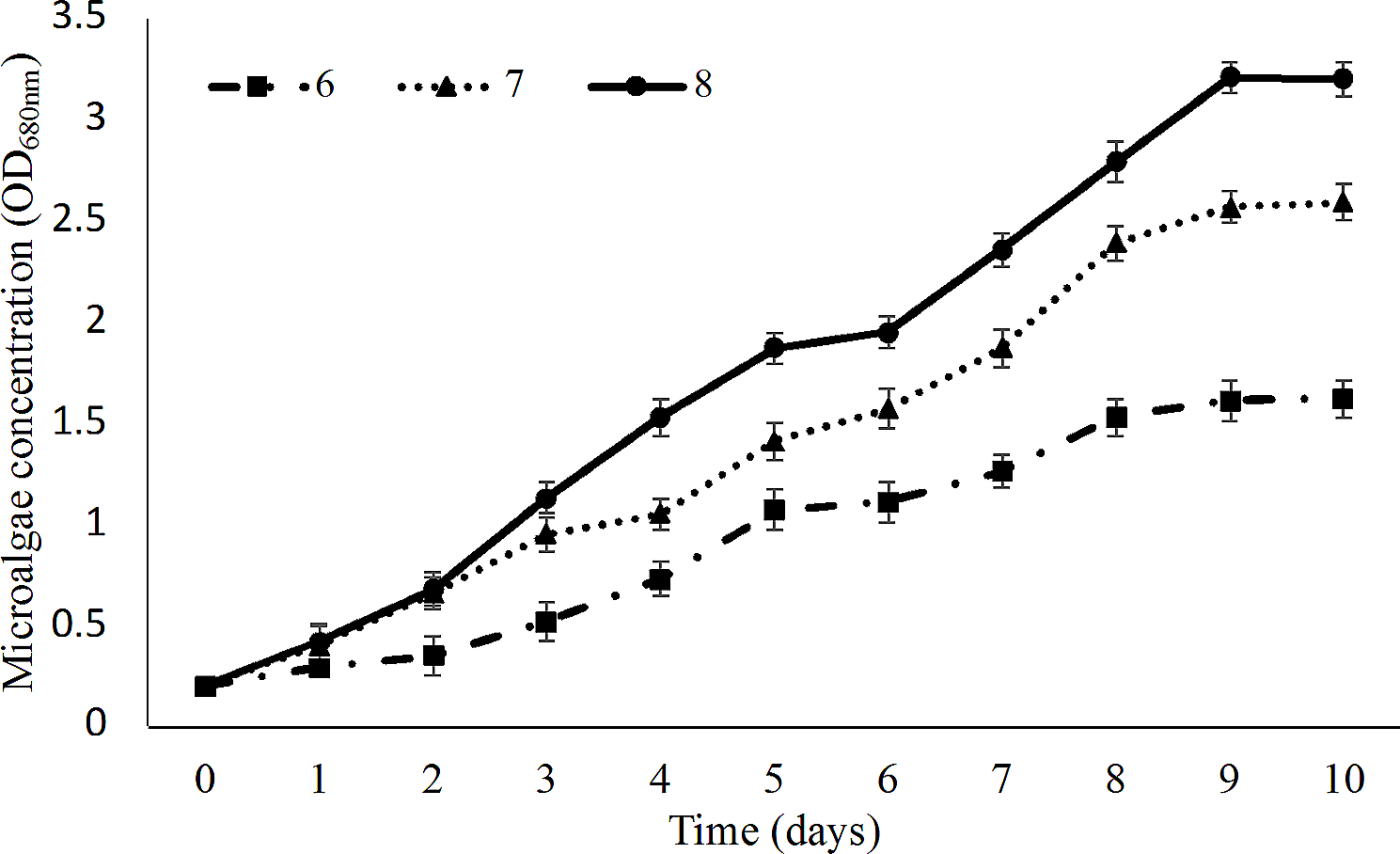
Growth curves of *Chlorella* sp. LH2 at different pH

Microalgae production was evaluated at different pH values. *Chlorella* sp. LH2 could grow in a wide range of pH including 6, 7, and 8 (Fig. 4). It’s reported that pH influences the activity of various enzymes of algae (Zhang et al. 2016). The optimal pH range of *Chlorella* is normally from 6 to 8.5 (Moss 1973). Our results indicated that an increase in pH value had favorable effect on biomass production. In the first day, the difference in term of algae concentration was not observed (*P* < 0.05). Since the second day, the growth rate of *Chlorella* sp. LH2 at pH of 6 was lower than that of pH of 7 and 8. The maximum productive biomass was achieved when *Chlorella* sp. LH2 was cultivated at pH of 8.

The susceptibility pH levels are species dependent (Kasai and Hatakeyama 1993). Some strains can tolerate at higher pH level of 9–11 (AlFadhly et al. 2022). In this study, the highest *Chlorella* sp. LH2 biomass yield was observed at pH of 8. Additionally, pH of hospital wastewater is slightly alkaline that is in the optimal range pH of microalgae (Akin 2016). This could result in saving related to operating cost in practical wastewater treatment.

### *Effects of the light/dark cycle on Chlorella* sp. LH2 *production*

**Fig. 5:**
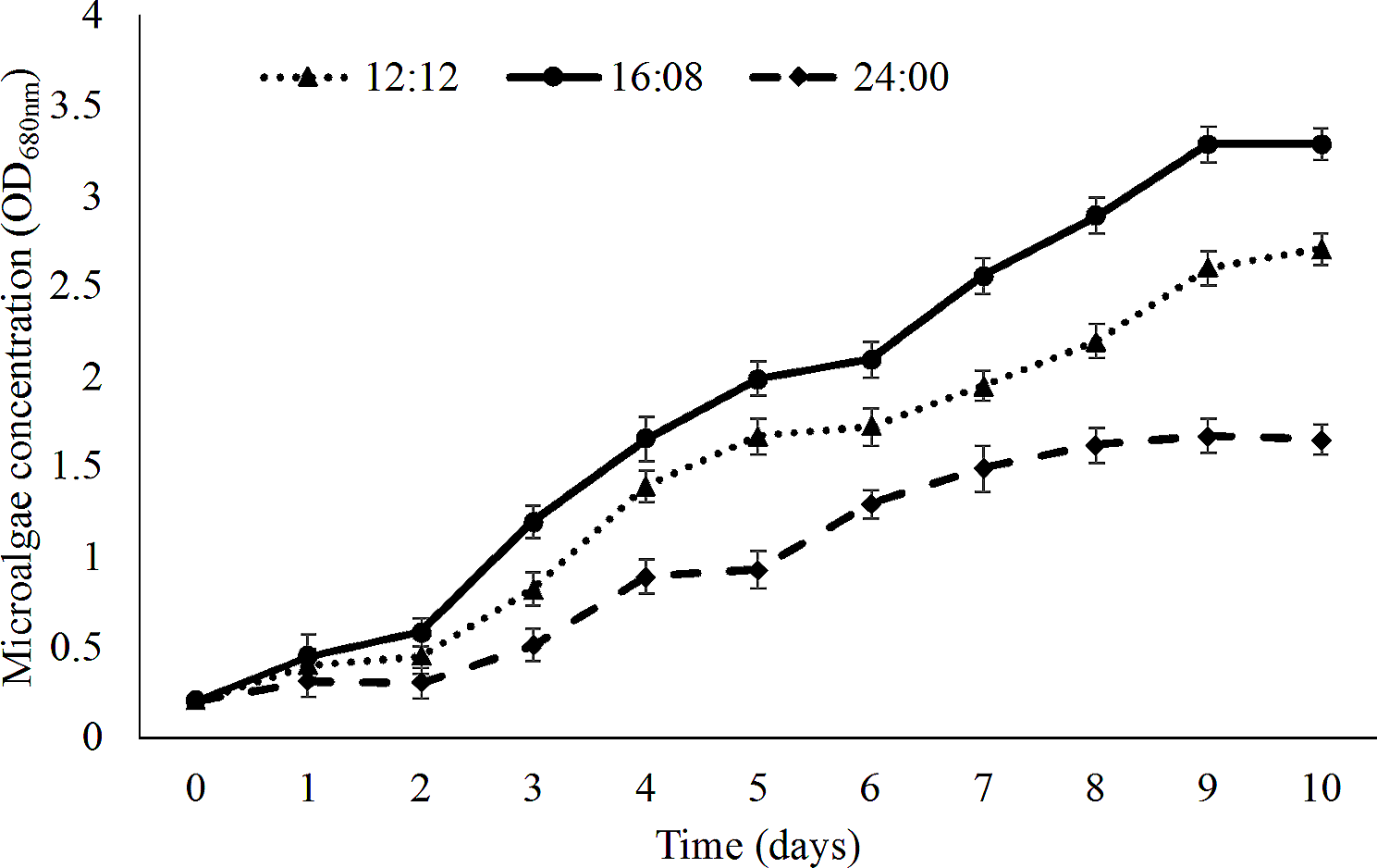
Growth curves of *Chlorella* sp. LH2 at different light:dark cycles

The effect of light:dark cycle on the *Chlorella* sp. LH2 growth at 30^o^C and pH of 8 is shown in Fig. 5. In the first day, the cell concentrations had minor difference among light:dark cycles, but no statistical difference. Since the second day, the cell density under light:dark cycle of 24:00 was lower than others. After 3 days, there was statistical differences in cell density among three light dark cycles.

Another factor contributed to the growth of microalgae yield is light:dark cycle (Gautam and Vinu 2020; Sun et al. 2022). Accumulation of cell material and cell increasing rely on light as a source of energy (Fu et al. 2012; Gong et al. 2014). The 18 and 24 h light cycles had the longer log phase than 12 h of light (Fig. 5). Additionally, the biomass yield of microalgae cultivated under 12 light cycle was lower than those of others. This may be caused by inadequate energy that promotes the growth during a long dark regime (Chauton et al. 2013).

The optimal light:dark cycle for *Chlorella* sp. LH2 growth in this study was 16:8. Light and dark phase in photosynthesis are necessary. While storing energy occurs during light period, utilization of these energy-pool molecules happens during dark period. It was indicated that ATP and NADPH, the light dependent phase compounds, are used in the dark phase to promoted the cell metabolism and biomass concentration of microalgae (Jacob-Lopes et al. 2009; Khoeyi et al. 2012). However, the cell biomass decreased under the 24:0 light:dark cycle. This is the result of photooxidation reaction inside the cells when excess light can not be absorbed (Phatarpekar et al. 2000; Richmond 2007). More light provides more energy for the development of microalgae, but the growth can be inhibited when this amount of light becomes too high (Simionato et al. 2011; Zhou et al. 2014).

### *Effects of the chlorine on microalgae production*

**Fig. 6:**
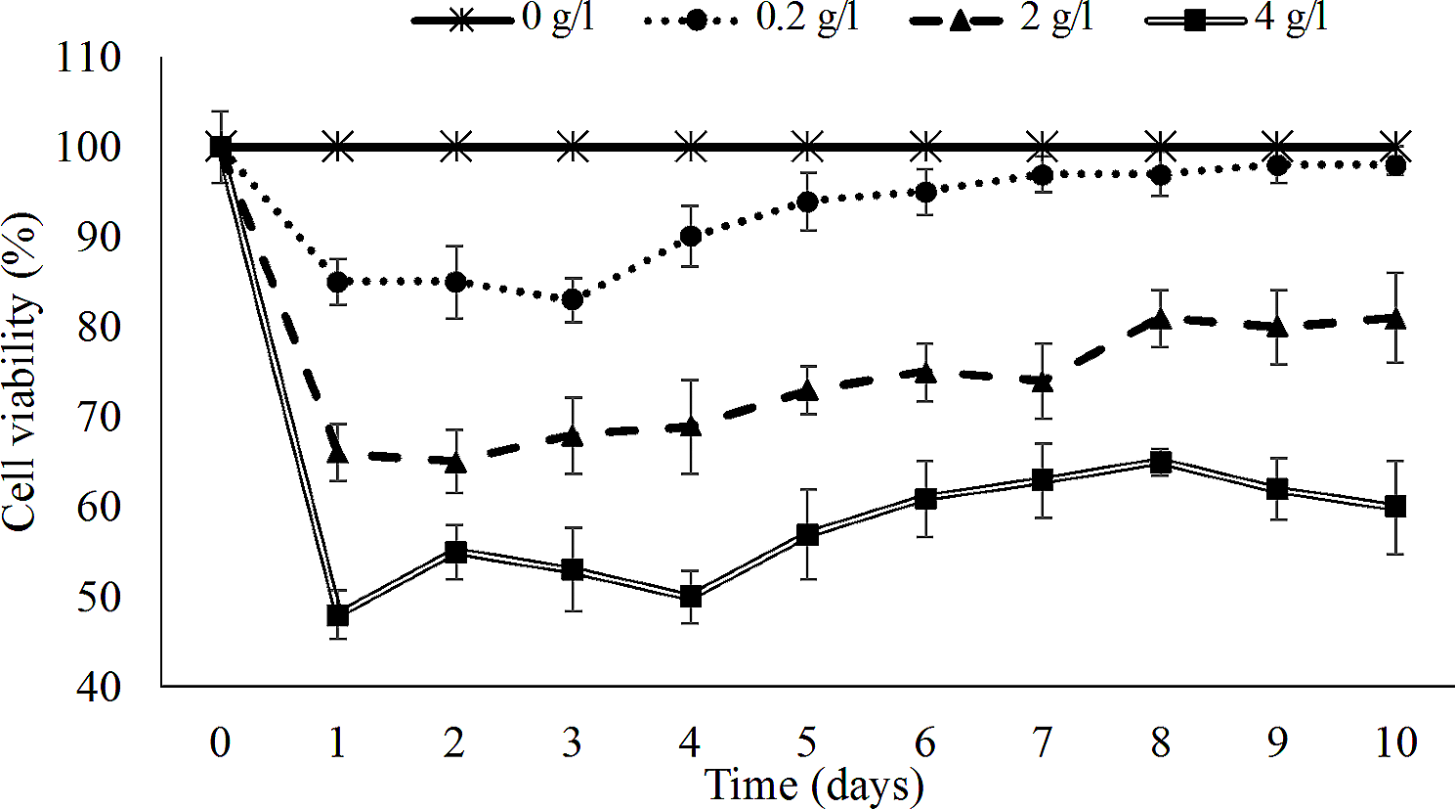
Chlorella sp. LH2 viability at different chlorine concentration

Chlorination is one of the methods to remove pathogenic microorganisms in water. However, disinfection by products can have a detrimental effect on organisms that live in the water bodies. The algal cell viability after being exposed to chlorine are presented in Fig. 6. The results revealed that increasing chlorine concentration led to the decrease of cell viability. Experiments were carried out under the optimal pH, temperature and light: dark cycle in HWW. The microalgae viability decreased when exposed to the chlorine concentrations of 0.2, 2, and 4 mg/l for the first 3 days compared to the control. Especially, the cell viability loss increased dramatically in the first day. Increasing chlorine dose promoted microalgae growth inhibition. The highest cell loss percentation was achieved at chlorine concentration of 4 g/l.

After the third day, the algae cultivated with chlorine concentration of 0.2 mg/l recovered and no statistical difference was observed. The concentration of cells exposed chlorine concentrations of 2 and 4 mg/l decreased dramatically and recovered slightly after 4 days. Chlorine causes cell death by disrupting cell wall and membrane, retarding respiration or metabolic process, inhibiting cell division, or damaging DNA (Denyer and Stewart 1998; Garoma and Yazdi 2019).

### *Pollutant removal by Chlorella* sp. LH2

Characteristics of wastewater before and after being treated with *Chlorella* sp. LH2 are presented in Table 1. The results show that HWW was rich in nitrogen and phosphorus. High levels of BOD_5_ and COD were observed. The ratio BOD_5_:COD was 0.77. When the BOD_5_:COD ratio of untreated wastewater is higher than 0.3, it indicates the wastewater is high biodegradability (Cossu et al. 2017). Thus using microalgae is one of suitable approaches to treat this wastewater. After 10 days, the performance for BOD_5_ removal was detected at 87.55%. The percentage of COD removal was 80.53%. The removal efficiency of T-N and T-P were 68.64% and 64.44%, respectively.

The eutrophication is able to be promoted when high content of N, P, and organic matter in HWW are discharged to receiving water bodies (Pauwels and Verstraete 2006; Paulus et al. 2019; Majumder et al. 2021). However, nutrients in the wastewater can be used to grow microalgae (Santos and Pires 2018). Therefore, microalgae can be cultivated in the wastewater as tertiary treatments to enhance the N and P removal (Ji et al. 2013; Morais et al. 2022). Indeed, the isolated strain of *Chlorella* sp. LH2 was able to thrive and remove the nutrients, both on N and P levels in HWW. The treatment had a positive effect on the decreasing COD and BOD_5_ as well. These results are consistent with the previous studies for the nutrients removal by *Chlorella* strains (Lau et al. 1996).

**Table 1:** Removal efficiency by *Chlorella* sp. LH2

Parameter	Initial concentration (mg/l)	Final concentration (mg/l)
**BOD**_**5**_	192 ± 8.62	23.9 ± 2.19
**COD**	243 ± 9.15	47.3 ± 5.71
**T-N**	49.2 ± 2.56	15.4 ± 3.27
**T-P**	2.4 ± 0.09	0.9 ± 0.12

### *Interaction between E. coli ATCC 8739 and Chlorella* sp. LH2

**Fig. 7:**
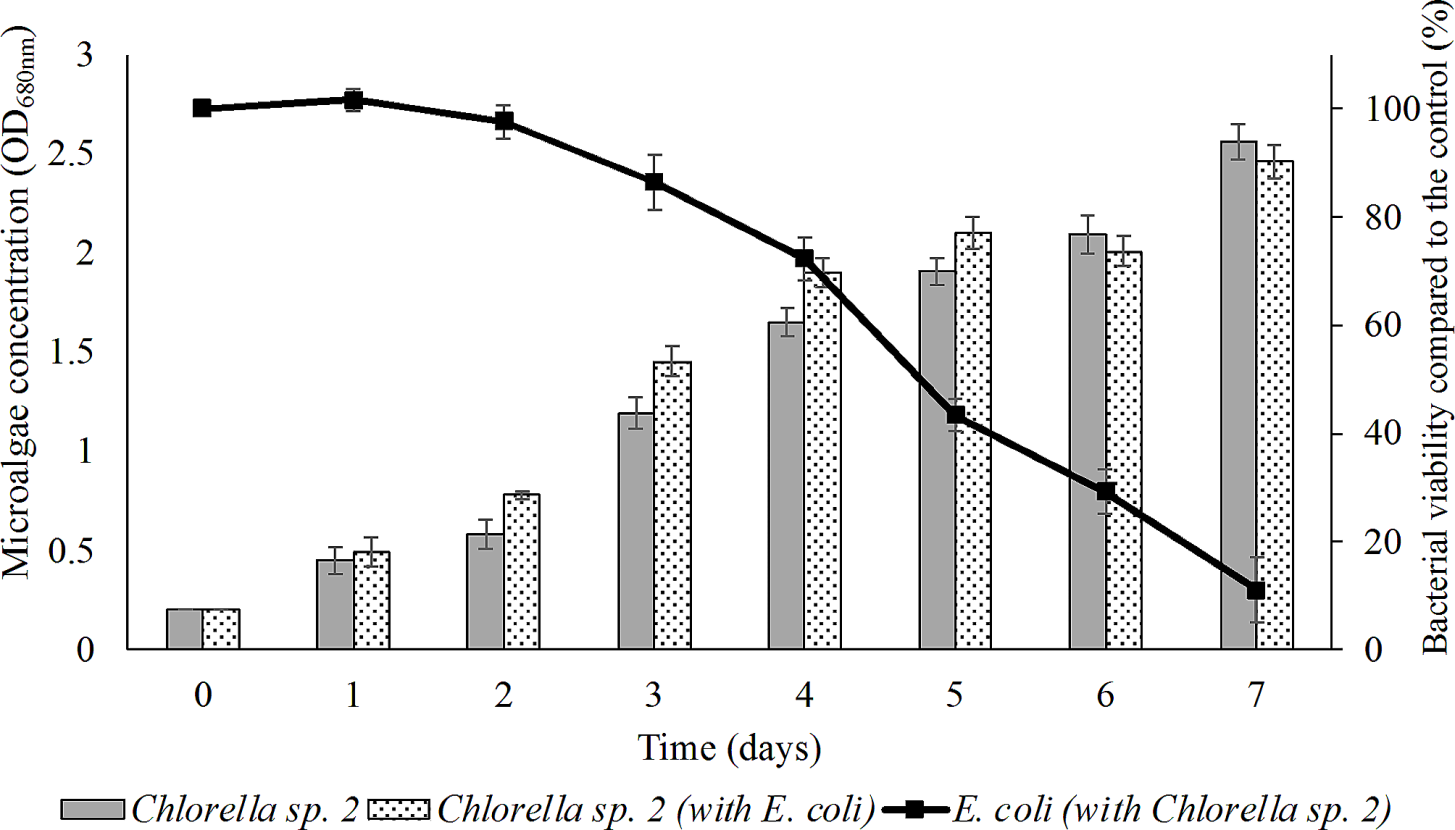
Interaction between *E. coli* ATCC 8739 and *Chlorella* sp. LH2

Various waterborne pathogens are found in effluents of HWW. Chlorination and ultraviolet (UV) disinfection are effective used methods to treat pathogens in wastewater. However, the resistance to chlorination or UV have been commonly detected (Rolbiecki et al. 2022). Previous studies were reported the restriction ability of microalgae on pathogens in wastewater (Christabel et al. 2019; Grossart and Simon 2007; Ribalet et al. 2008; Shaima et al. 2022). As shown in Fig. 7, the growth of *E. coli* ATCC 8739 was observed both in the absence and presence of *Chlorella* sp. LH2. In the first 2 days, *E. coli* ATCC 8739 thrived in hospital wastewater in both conditions with and without microalgae. Bacterial removal were not detected. The variety was observed at the 3rd day. The concentration of *E. coli* incubated with *Chlorella* sp. LH2 was decreasing from the 3rd day to the end of experiment. The inhibition effects of *Chlorella* sp. LH2 on *E. coli* ATCC 8739 was retarded in the first 3 days could be due to low algae concentration. Microalgae can secrete antibiotic compounds that are low concentration at the lag phase of microalgae growth (Grossart and Simon 2007). The removal efficiency at the 7th day gained 88.92%. Bacterial growth was achieved by using nutrients in wastewater. However, microalgae development since the third date of cultivation inhibited bacterial growth. The competition nutrients between bacteria and algae and secreted antibiotic compounds from algae, as well, may suppress bacterial growth (Lekunberri et al. 2012; Qu et al. 2014).

In the first stage of development, the growth of *Chlorella* sp. LH2 was stimulated by *E. coli* ATCC 8739 (*P* < 0.05). This may the results of symbiotic relationship between algae and bacteria. Carbon dioxide from bacterial respiration can stimulate algal growth. Laterly, the removal of *E. coli* was observed after the thriving of *Chlorella* sp. LH2. In addition, there was no statistically difference between 2 conditions (with and without *E. coli*) in term of algal biomass in the 6th and 7th day. The mode of restriction may be caused by the increase of oxygenation (Ansa et al. 2015) and pH elevation (Higgins and VanderGheynst 2014) during microalgal growing. Additionally, the demand nutrients related high density may shift the cooperation to competitive relationship between *Chlorella* sp. LH2 and *E. coli* ATCC 8739 (Mayo and Noike 1996; Ansa et al. 2015; Žitnik et al. 2019).

## Conclusions

This study provides a proof in capabilities of *Chlorella* sp. LH2 strain in growing and reducing nutrients in hospital and cocoon wastewater. The productive was no statistical difference among three culture media including BG11, hospital, and cocoon wastewater. *Chlorella* sp. LH2 that isolated from the cocoon wastewater could thrive in hospital wastewater as only nutrient source. The growth depends on culturing conditions including temperature, pH, and light: dark cycle. Microalgae growth was suppressed by chlorination of 2 and 4 mg/l. BOD_5_:COD ratio of untreated HWW is 0.77 that exhibited high biodegradability. Efficiency of this algae for nutrients removal was detected. The performance of COD and BOD_5_ removal after 10 days were 80.53% and 87.56%, respectively. The removal of total nitrogen and total phosphorus were 68.64% and 64.44%, respectively. The growth of *E.coli* ATCC 8739 was inhibited by *Chlorella* sp. LH2. More comprehensive studies are required to understand the interactions and mechanism in elimination bacterial pathogens of *Chlorella* sp. LH2.

### Electronic supplementary material

Below is the link to the electronic supplementary material.

Supplementary Material 1

Supplementary Material 2

## Data Availability

The datasets generated during and/or analyzed during the current study are used in this manuscript are included in this document.

## Abbreviations

BMW: Biomedical waste
BOD: Biological oxygen demand
COD: Chemical oxygen demand
ECs: Emerging contaminants
HWW: Hospital wastewater
T-N: Total nitrogen
OD: Optical density
T-P: Total phosphorus
WW: Wastewater
