# Supplementary material for: Potential of hospital wastewater treatment using locally isolated Chlorella sp. LH2 from cocoon wastewater
Source: Bioresour Bioprocess. 2024 Apr 6;11(1):35. doi: 10.1186/s40643-024-00748-6 (PMC10998823; doi:10.1186/s40643-024-00748-6)
Supplement: Supplementary file 1 — Supplementary Material 1 [file 40643_2024_748_MOESM1_ESM.docx]

**Potential of hospital wastewater treatment using locally isolated *Chlorella* sp. LH2 from cocoon wastewater**

Ethics approval: Not Applicable
