# Supplementary figures and images for: Potential of hospital wastewater treatment using locally isolated Chlorella sp. LH2 from cocoon wastewater
Source: Bioresour Bioprocess. 2024 Apr 6;11(1):35. doi: 10.1186/s40643-024-00748-6 (PMC10998823; doi:10.1186/s40643-024-00748-6)

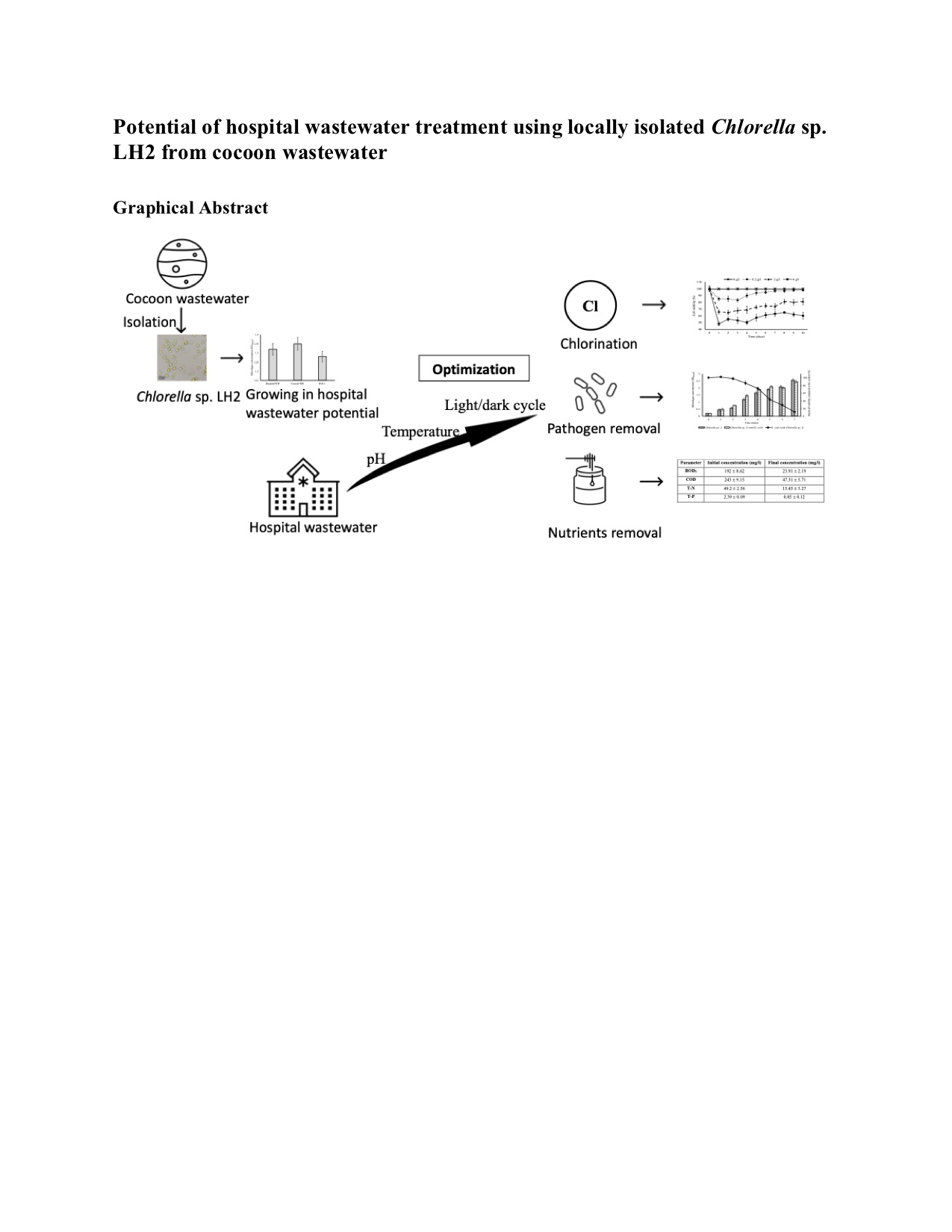

Supplement: Supplementary file 2 — Supplementary Material 2 [file 40643_2024_748_MOESM2_ESM.jpg]
